# The Impact of Periodic Formative Assessments on Learning Through the Lens of the Complex Adaptive System and Social Sustainability Principles

**DOI:** 10.7759/cureus.41072

**Published:** 2023-06-28

**Authors:** Sanjukta Sahoo, Amit P Tirpude, Prabhas R Tripathy, Manisha R Gaikwad, Sanjay Giri

**Affiliations:** 1 Anatomy, All India Institute of Medical Sciences, Bhubaneswar, Bhubaneswar, IND; 2 Anatomy, All India Institute of Medical Sciences, Raipur, Raipur, IND; 3 Plastic Surgery, All India Institute of Medical Sciences, Bhubaneswar, Bhubaneswar, IND

**Keywords:** feedback, sustainability, adaptive, assessment, formative

## Abstract

Purpose: Medical institutions in India have employed different periodic formative assessment (FA) methods with variable impact. The formative evaluation must incorporate feedback to improve learning. Formative assessment has helped inexperienced students apprehend their weaknesses, make choices, prepare for summative exams, and allow teachers to identify regions wherein students may also need aid. This study attempts to quantify the impact of the weekly, monthly, and semester formative assessments and view it retrospectively through the lens of complex adaptive systems and social sustainability principles.

Methods: We used a post-exam survey and statistical analysis to compare the students' performance between the timely periodic formative assessments in a competency-based curriculum. The cohort consisted of 2018 (semester), 2019 (weekly), and 2020 (monthly) first-year medical students. Cronbach alpha, spearman's correlation coefficient, descriptive statistics, and repeated measure analysis of variance were used to explore the reliability and relationship between formative assessment and summative scores of each cohort and find any significant difference. The authors also analyzed the accordance between the FA exam performance and students' perceptions, deduced broad themes, and discussed the appropriateness and feasibility of students' suggestions for changes.

Results: A significant correlation was found only between the weekly formative assessment and summative scores (r=0.74, p=0.01). The analysis of variance established significant differences between all summative scores of respective periodic formative assessments. The weekly formative assessment showed the highest mean summative examination scores. This study helped comprehend preclinical students' apprehension after the intervention of periodic formative assessments. The students found this intervention helpful in driving and detecting gaps in learning but preferred focused feedback, clinically oriented practices, and countered mental health issues.

Conclusion: The continuous periodic formative assessment model had a valid educational impact but was not sustainable according to social sustainability principles. A complex adaptive framework can be utilized to make it sustainable.

## Introduction

Formative assessment (FA) allows you to track student progress in real-time and change the course curriculum and instruction as necessary. FA manages misconceptions, struggles, and mastering gaps along the way and examines how to close these gaps. It consists of tools for mastering and can even bolster students’ competencies to take possession of their gaining knowledge when they apprehend that the aim is to enhance learning and no longer follow the final marks [1].

Feedback profoundly impacts a student's learning, performance, and efficacy of the FA [2]. Feedback within the formative assessment must be 'unique, correct, timely, transparent, targeted upon the manageable and expressed to encourage a person to mirror his learning and experience the need to alternate [3].

Progressive institutions are exploring innovative methods in continuous formative assessment. Scheduled development exams verify a student's depth of learning in the preclinical setting. It's interesting for science educators and college students alike to perceive which modifiable elements best benefit examination preparation [4]. Imposing any new intervention in formative assessment wants to be strongly supported with the resource of seriously analyzed proof of college and students' achievements. The intervention needs an appropriate environment, constant motivation, determination, correct strategies, faculties' training, dissemination of facts to college students, proactiveness, and steering of the medical education unit for integration into the curriculum and simultaneous evaluation [5].

The criteria for the testimony of an assessment are validity, reliability, feasibility, educational impact, and timely feedback [6]. Structuring the implementation of a couple of checks on more than one content material region using a couple of examiners using numerous tools in various settings in the proposed quarter model will improve the reliability and validity of internal assessment and its acceptability [7].

Assessment practices should send the right cues to students approximately what and how they should be mastering. Extra regularly than not, students perceive wrong indicators; thus, it is essential to examine students' perceptions of the purposes of assessment, the relationship between assessment, the assumed nature of what is being assessed, and how different assessment formats impact learning [8]. 

We compared the student’s performance between the traditional (semester) and the interventions of periodic FA in a competency-based curriculum. A convergent mix model design through a post-exam survey where broad themes were explored was used, and the combined evidence generated examined the impact on learning. 

Competency-based medical education may require frequent FA, but the sum of all FA does not result in reliable measures. The slow development in assessment fruitfulness can be attributed to the openly reductionist approach [9]. The emerging trends in innovative anatomy teaching now demand a constant look at mental health and equitable access for all students.

This lack of desired holistic achievement or unsustainability may be due to the assumption and interpretations based on linear hierarchical cause-and-effect relationships by policymakers, planners, and researchers. FA creates complexity challenges as it offers a range of methodological problems. It requires ongoing and well-timed evaluation and refinement to consider the impact of the instructions. Furthermore, it becomes feasible to measure consequences not only after the execution of an approach but also the issue of when to conduct it to consider the success of a strategy [10]. Adopting an integrated and collaborative approach involving all stakeholders is imperative to ensure equal access to education and achieve sustainable development goals [11].

The complex adaptive system (CAS) approach is a way of thinking and analyzing things by recognizing complexity, patterns, and interrelationships between multiple agents in a system, e.g. school. It challenges the simple linear cause-and-effect assumption such that the interventions of instructors and the conduct of FAs improve overall students' performance. It instead sees health care and other systems as a dynamic process in a system where the interactions and relationships of different components affect and shape the system. Agents (teachers, students, etc.) in any system can be all components of that system and interact and connect with each other in an unpredictable and unplanned way. Interactions of agents within the system begin to form emerging patterns which, in turn, feedback into the system and further influence the agent’s interactions. CAS can adapt multiple agents interacting in a non-linear fashion. Through adaptation, they judge and operate in a situational context. These interactions between top-to-bottom macro and micro-scale organizations generate tension, creating core complexity. The system self-organizes itself to emerge a coherent global pattern that typically cannot be predicted from agents' behavior in isolation. The CAS can self-organize into a critical state where feedback loops work to amplify some small perturbation into a significant systemic effect, or it may want to minimize deviation to remain stable [12]. CAS provides a framework for categorizing and analyzing the knowledge and agents and suggesting a complete picture of forces affecting change and the possibility for a change. It provides a dynamic picture of shared influences on the intervention [13]. The CAS framework can inform us how to introduce, sustain and implement interventions [14]. CAS sees the organization as a living system where information flow, participation, and resilience are vital aspects helping to develop more effective strategies [15]. Many studies use CAS as an analysis approach, an explanatory device [16].

There are several essential aspects of adaptive capacity in a social system, such as trust, the capacity to self-organize, and shared meaning [17]. Woodruff proposed a model of two principles of intrinsic motivation: agents contribute to the task and the absence of excessive central control, which offers opportunities for continuous improvement and may provide a comprehensive understanding of how education and training activities may lead to more effective behavioral change and system improvement [18].

When the agents are adapting, the system is unsustainable or evolving. This phenomenon is undesirable for perpetuity, and the final aim is to find a mechanism to cease the system from being sufficiently adaptive. This process has led to the generation of social sustainability principles (SSP). Social sustainability is a condition that is a positive, life-enhancing condition within communities and a process that can achieve that condition [19]. Sustainability wants that people are not subjected to structural obstacles such as health so they will fall ill, influenced that will disallow their participation in shaping the system, competence that will disallow their individual development, impartiality that people are exposed to partial treatment, and meaning-making that is they are disallowed to express their thoughts about the process [17]. In this study, the system level describes the conduct and functioning of the FA practice in our institution, and the success level is defined as achieving sustainability and competence without affecting students' social and mental conditions. Concrete examples of the application of CAS and SSP framework are difficult to find due to the paucity of empirical research, but some studies are conducted to investigate leadership styles, organizational change, team dynamics, and sustainability [20]. Authors, in retrospection, viewed the practice of FA through a system of complex adaptation perspective and whether it withstands SSPs. Finally, we attempted to contribute and inform the established literature on FA.

Organizational context - The Anatomy course at All India Institute of Medical Sciences Bhubaneswar (AIIMS BBSR) is competency-based. Each batch comprised a hundred students. The assessment format used in the study includes written examinations such as multiple-choice questions (MCQ), short answer questions (SAQ), essay questions, and objectively structured spotters and viva in practicals.

## Materials and methods

After the periodic informal internal review of summative assessment (SA) and formative assessment evaluation, the medical education department AIIMS BBSR recommended weekly FA for the 2019 batch. The cut-off marks for eligibility to appear in the professional exam were the average of total marks obtained in n-3 (to exclude the three lowest marks obtained) numbers of tests conducted. The 2020 batch FA was conducted monthly after the request from students. The cut-off marks were n-1 (to exclude the single lowest marks obtained) numbers of tests. Here 'n' is the number of tests conducted for qualification for summative examination. The 2018 batch went through the traditional quarterly FAs. Table 1 illustrates the demographic characteristics of the participants in FAs and the post-exam survey.

**Table 1:** Demographics of participants in the FA and post-exam survey FA - Formative Assessment

Frequency						
Variables	Batch 2018		Batch 2019		Batch 2020	
	FA	Survey	FA	Survey	FA	Survey
Total No. of respondents	102	51	106	59	131	69
No. of Male respondents	64	28	78	43	73	48
No. of female respondents	38	23	28	16	58	21

All authors accepted the convergent mix model design proposal where quantitative and qualitative data are collected, analyzed, and compared to see if the data confirms or disconfirms each other. The quantitative data were the FA's, summative examination scores, and the frequency distribution of student perception of FA conducted as a post-exam survey of 2018, 2019, and 2020 batches. The qualitative data were the broad themes generated from the free text responses. The institutional ethics committee approved the study vide no. T/IM-NF/Anatomy/21/132. All students and faculty gave consent to participate in the study. Experienced teachers used the competency standard of must-know to set the question papers. The topics and essential questions were displayed on the notice board appropriately. One hour was allotted for weekly and monthly FA. Three hours were allotted for semester examinations. The written papers were randomized before correction. The students consented to submit an online Google form of their perceptions before submitting the survey. After thorough deliberation, all concerned faculty agreed to adopt the original article questionnaire of Preston et al. 2020 with some modifications after revalidation for the survey (Table 2) [21].

**Table 2:** Survey on perceptions of periodic formative assessments The survey questions were divided into two sections. Section A: The first section included questions about participants’ demographic characteristics (year of study, enrolment status, and gender). Section B: The second section contained questions related to participants’ perceptions of the assessment.

Survey Questions					
Ques 1: How accurately does weekly/monthly or semester FA reflect your effort into learning?					
1=least accurate	2= moderately accurate		3=most accurate		
Ques 2: How accurately does weekly/monthly or semester FA reflect your knowledge of the content material?					
1=least accurate	2= moderately accurate	3=most accurate			
Ques 3: How useful is weekly/monthly or semester FA in driving your learning?					
1= very useful	2=useful		3=neither useful nor useless	4=not useful	5=not at all useful
Ques 4: Overall, how would you rate the assessment load in each periodic FA in your current year of study?					
1=very light	2=light		3=neither light nor load	4=heavy	5=very heavy
Ques 5: Which description best reflects how you perceive assessment? (Tick only one)					
(a) Assessment helps me identify current gaps in my learning					
(b) Assessment enables me to address gaps in my learning					
(c) Assessment quantifies my level of knowledge and/or competence					
Ques 6: We recommend weekly tests for formative assessment.					
1=strongly agree	2=agree		3=neither agree nor disagree	4=disagree	5=strongly disagree
Ques 7: We recommend monthly tests for formative tests.					
1=strongly agree	2=agree		3=neither agree nor disagree	4=disagree	5=strongly disagree
Ques 8: Formative Assessment gives an idea about the questions or the patterns to be asked.					
1=strongly agree	2=agree		3=neither agree nor disagree	4=disagree	5=strongly disagree
Ques 9: We get an opportunity for optimum feedback to improve our performance.					
1=strongly agree	2=agree		3=neither agree nor disagree	4=disagree	5=strongly disagree
Ques 10: The following methods are useful for formative assessment.					
A) Written only					
1=strongly agree	2=agree		3=neither agree nor disagree	4=disagree	5=strongly disagree
B) Viva only					
1=strongly agree	2=agree		3=neither agree nor disagree	4=disagree	5=strongly disagree
C) Practical					
1=strongly agree	2=agree		3=neither agree nor disagree	4=disagree	5=strongly disagree
D) All of the above					
1=strongly agree	2=agree		3=neither agree nor disagree	4=disagree	5=strongly disagree
Ques 11: If you could change one thing about assessment, what would that be? You are free to write about your reservations about the current system and the questions asked in the survey (free text).					

The Google form data was divided into two broad sections: demographic characteristics and the perception about the level of accuracy of each of the current suite of assessment tools in reflecting the effort they put into learning, knowledge of content material, and the usefulness of on-course assessment. Participants were also required to select the best descriptor free-text comments (1 out of 3) of their perception of the assessment and the one thing about the assessment that they would want to change. The quantitative data were trimmed and analyzed using MS Excel. We used Cronbach alpha, spearman's correlation coefficient, descriptive statistics, and repeated measure analysis of variance to explore the reliability and relationship between FA assessment and summative scores of each cohort and find any significant difference between summative scores of periodic FA's, respectively. The authors also analyzed the accordance between the FA exam performance and students' perceptions. After using ‘R’ studio (RStudio, Inc., Boston, MA) to generate insights about word association from free text data, they deduced broad themes manually by pen and paper notes. We also discussed the appropriateness and feasibility of students' suggestions for changes. All the open-ended questions were examined by an inductive qualitative method through open coding and discovering emergent themes. All authors backcasted the whole scenario through CAS and SSP principles.

## Results

Table 1 figures are coherent with the cohort's profile and representative of the sample participants. The Cronbach alpha coefficients were 0.65 and 0.67 for questions with three and five options. The Spearman's correlation coefficient between FA's and the SA scores were 0.44, 0.74, and 0.48 for the semester, weekly and monthly assessments. Only weekly FA and SA scores correlated highly significantly. Tables 3-4 present the mean±sd registered for each response for each cohort. Irrespective of the periodic nature of FA, the authors assumed each cohort's final professional exam score as the average performance score of each student who attended the FA. Table 5 shows the significant difference between each cohort's mean summative exam scores (p-value <0.0001) after applying the repeated measure ANOVA using the non-parametric Dunn's test. The mean score of the weekly exam is more than the semester and monthly FA. The major themes generated from free text responses of the 2018 batch were to increase the frequency of FA, prepare syllabus in consultation with students, relax eligibility criteria for professional examination and incorporate more MCQs. The 2019 batch requested to incorporate more MCQs, relax eligibility criteria for a professional examination, increase anxiety, stress, and fear, have a sympathetic marking scheme, and remove short notes. The 2020 batch reported mental health issues and requested to reduce lectures.

**Table 3:** Perceptions of questions 1-6 (Mean ±SD)

Variable	Q.1	Q.2	Q.3	Q.4	Q.5	Q.6
2018 Semester	1.84±1.32	1.72±.23	2.08±1.93	2.24±2.0	1.68±1.32	4.64±4.19
2019 weekly	1.96±1.49	1.98±1.46	1.96±1.75	3.72±3.42	1.55±1.22	4.15±3.81
2020 monthly	2.26±1.75	2.22±1.73	1.94±1.61	3.59±3.34	1.62±1.28	4.19±3.87

**Table 4:** Perceptions of questions 7-10 (Mean ±SD) SD-standard deviation

Variable	Q.7	Q.8	Q.9	Q.10			
				A	B	C	D
2018 Semester	3.92±3.61	2.44±2.29	2.68±2.44	2.96±2.74	3.56±3.32	3.0±2.87	2.28±2.09
2019 weekly	3.11±2.60	2.08±1.62	2.59±2.34	2.52±2.21	3.33±3.04	3.10±2.80	3.87±2.84
2020 monthly	1.54±1.38	1.69±1.28	1.8±1.38	2.55±2.35	3.36±3.02	3.06±2.79	1.58±1.44

**Table 5:** Differences between summative professional exam scores

Variable		Mean± SD		Comparison	Repeated measure ANOVA with non-parametric Dunn's test	
Semester		101.91±20.52	Semester vs Monthly			P<0.001
Weekly		120.39±17.59	Semester vs Weekly			P<0.001
Monthly		111.70±13.34	Monthly vs Weekly			P<0.05

## Discussion

The Cronbach alpha coefficients of 0.65 and 0.67 for questions with three and five options indicate our modified post-exam survey's acceptable reliability and internal consistency. The authors found a disparity between the FA exam attendees and those who filled out the Google form (Table 2). However, these can be considered representatives of the desired population. We have not calculated the accuracy of each assessment tool. This paper only attempts to examine the impact of periodic FA on learning while keeping the method constant. The significance found in the positive correlation coefficient and repeated measure ANOVA (Table 5) between FA and SA exam scores in weekly FA demonstrates that under a fixed format of the examination, it can test the breadth of a student's knowledge and be an impactful modifiable factor. However, students indicated a significant load in the reflections about weekly assessments (Table 3, question number 4).

Thirty-seven and thirty percent of students in the 2019 batch reported the assessment load as very heavy to heavy, respectively. Thirty-four and twenty-seven percent of 2020 batch students reported the assessment load as 'very heavy' and 'heavy' respectively. Sixty percent of 2018 batch students reported the assessment load as very light. The majority of students strongly disagreed with the conduction of weekly tests. The students feel depressed, anxious, and fearful in weekly FA despite essential competencies likely to be asked in the examination being pre-notified to students as expected in reflections: "Stop conducting weekly assessment and if conducted don't keep criteria to secure 50% in n-1. You don't know how much havoc you have already created in the lives of some students who even did not know how it feels to be depressed. Would you like to see your son/daughter in the same condition? If you can then conduct an exam with the criteria of securing 50% marks to be able to sit in a final exam". Another student raised concern: "The problem of having weekly exams is students are constantly put under stress, one may argue that it's a medical college so students are supposed to feel the stress, but that's not the case, the number of students going into depression due to exams in this college is increasing at an alarming rate". The assessment load in monthly FA's was also heavy, but in perceptions, we didn't find any significant complaint: "More frequent exams don't improve learning. It doesn't give time to build a deep understanding of the subject. It makes the goal to get marks rather than understand the subject. Understanding the subject requires reading things that will never come in exams. Frequent exams make it difficult to study things other than what will come in the exam". This reinforces the observation that performance in specific assessments may not indicate the perceived relevance of evaluation [21], and assessment is a common source of stress and anxiety [22]. This issue also aligns with the 2021 Educause Horizon report stating the need to rethink the student experience to meet students where they are, utilizing emerging technology tools and policies to ensure wellness and pre-empt emergency mental health situations [23].

The maximum number of students have chosen the option of moderately accurate to reflect upon the accuracy and usefulness of periodic assessments in terms of effort put into learning, knowledge of content material, and as a driver of learning. The reflections noted mostly favored the monthly assessments. This midway approach of students hints at some hidden aspirations about FA.

Surprisingly, in free text responses even when we kept the examination's structural format constant, students freely expressed their mixed views favoring MCQ-based exam: "Please introduce only MCQ instead of recent exams. It's not useful at all. Only MCQs test real knowledge. Some institutions are doing very well in postgraduate entrance exams than ours because of this only". A student of 2018 reflected: "Please take more MCQ-based exams and keep a suitable gap between two exams". This perception matches the view that MCQs better reflect their efforts and help in the application of clinical scenarios [21]. Regarding the weightings of FA, a student of 2019 reflected: "The load is too much… It should be reduced, and the same weightage should not be given to weekly assessments and end-semester exams". This acknowledges the student's perception that periodic FA did not always reflect the time and effort put in and maybe their perception about filling the learning gap [21]. This also shows the concern of students about the actual longitudinal benefit of FAs concerning graduation and qualifying examinations than about the pattern of questions.

More than 50% of students have acknowledged the weekly and monthly FA as an approach that helps them to identify the current knowledge gaps in learning. However, significant gaps in student learning may result as not every student is the same and may need extra support. Helping students form connections and identifying gaps is stressed. An early build-up of a sense of community and belonging, providing an opportunity to interact, and encouraging study group formation can help set up connections. Identifying the extent of the gap may be possible by providing a concept test or other low stake assessments and guiding them to overcome challenges by sharing experiences openly on different platforms and peer support [24]. All institutes need to inculcate this adaptive approach.

Students considered feedback as helpful if mentored one-to-one. The evidence in the literature demands detailed, personalized, focused, and constructive feedback that can increase engagement and student learning [25]. One student reflected: "Students should be taught to present an answer properly rather than writing many unnecessary things". This aligns with the view that students valued feedback when it included focused suggestions on improvement in the form of written comments, including examples and explanations in the case of written assessment pieces [26].

Students were comfortable with the marking scheme but wanted it not to be an eligibility criterion for the final professional examination. Using explicit marking criteria, knowledge and familiarity of the rubric, and creating rubrics in consultation with students can improve academic performance and enhance students' satisfaction with feedback [27]. 

The majority of students in free text responses indicated that FAs are relevant to them if the full focus is given to clinical scenario-based MCQs, which will be helpful to them in professional examinations and clinical practice. This observation hints at the academic awareness of preclinical students and the demand for 'authenticity' in assessment [28].

The faculty identified and categorized the demands of students into realistic, feasible, and unrealistic. The feasible and realistic suggestions, such as stopping the weekly FAs, incorporating more clinical scenario-based MCQs, notifying important expected questions, and providing more self-study hours, were implemented. The request for a sympathetic marking scheme and not to consider the performance of FAs for eligibility to appear in the professional examination was rejected as that may undermine the value of FA amongst students.

Chaudhary et al. (2019) demonstrated better student performance in weekly FA and recorded high learner satisfaction on the Likert scale [29]. Students' comments that it provided a safe environment with faculty assistance need scrutiny. The non-significance of monthly and semester exams must also be matched with perceptions.

Santra et al., in their non-interventional study, observed a non-linear significant partial direct correlation (r = 0.26, p<0.01) between the FA and final summative examination performance scores [30]. They hinted at other possible independent variables influencing the final result, such as learning styles, demographics, and entry qualifications. They also concluded that the yearly performance of students is reflected in FA, and it can generally be presumed to be a predictor of final summative examination results. This influence of some independent variables may also play an essential role in the mismatch between weekly FA scores and perceptions.

Before the implementation of periodic FA, fifty percent of preclinical faculty had basic training in medical education, but training in quality assurance in assessment practices is exclusive. The intrinsic motivation was present to implement the program but technical expertise is doubtful. The decision of implementation lacks reflections from faculty or an authentic source. The experiences shared about conduct were intradepartmental and lacked peer norming, which decreased the capacity to adapt for a common purpose. The involvement of students in this process was minimal except for common online feedback. The peers of the student, supporting staff, hostel companions, and parents can help process mapping discussion around feedback and will foster openness, autonomy, and proportional regulation. Shortcomings of these initiatives naturally belittle the FA process and limit the development of feedback loops and mutual trust among teachers and students. Predominantly weekly FA's has exhibited an emergent self-organizing pattern where the students find it challenging to their mental health, non-participatory in shaping it, and over-competitive. This observation aligns with the council's predictions on competency-based medical education [9]. In India, students come from different socio-economic backgrounds and inherently have issues with self-identification. This diversity can be utilized for coadaptation, preventing unintentional partiality. This model of FA does not emphasize inclusion which offers an opportunity to reform medical culture [31].

The Cronbach alpha indicates the internal consistency and reproducibility of the periodic FA scores. FA's were intended for the assessment of learning and acted as a development exercise for students and it was a well-informed decision for the students. All the FA in anatomy exploited all structural formats in written papers aligned to blueprinted, mapped to learning outcomes in a revamped curriculum, and set by experienced faculty after departmental discussion. The exams were held in an impartial environment under the observation of interdepartmental invigilators. All examiners were familiar with the test format and examinees understood the regulations. The correlation between FA's and summative examination scores was positive and the intended impact on learning was observed. The theory and practical examination counters were double-blinded and average scores were accepted unbiasedly after providing enough opportunities to students. FA's intention was clear, and a criterion reference of 50% was used as a qualifying criterion for professional examinations. The robustness of this format, a shred of evidence in the form of online feedback, and a positive correlation between FA and SA scores demonstrate this model as a valid one. The educational impact of this model is affirmative but at the cost of students' mental health. The model is cost-effective and has affordable resource intensiveness. We conclude that this model is not sustainable unless a significant revision is attempted and, in the future, it is operated in an adaptive system model.

Recommendations

In the future, the impact of periodic formative assessments in the Indian context may be studied by triangulation of findings of psychometrics, using an appropriate qualitative tool, and adaptive approach.

Limitations

Some participants have not submitted their responses to the feedback questionnaire. This study's findings are not generalizable to other settings with different teaching and assessment methods. This study reflects findings in an Indian context.

## Conclusions

This study helped comprehend preclinical students' apprehension after the intervention of periodic FAs while keeping the assessment methods constant. The students find this intervention helpful in driving and detecting gaps in learning, but preferred focused feedback, clinically oriented methods, and countered mental health issues. The practice of focused feedback is a necessary step to ensure the quality of the assessment process. The model of a complex adaptive system and social sustainability principles can be incorporated to fulfill the purpose of assessment and make it sustainable.

## References

[1] Trumbull E, Lash A (2013). Understanding Formative Assessment: Insights From Learning Theory and Measurement Theory. Published online.

[2] Fraser BJ (1987). Identifying the salient facets of a model of student learning: a synthesis of meta analyses. Int J Educ Res.

[3] Jenkins JO (2010). A multi‐faceted formative assessment approach: better recognising the learning needs of students. Assess Eval High Educ.

[4] Bauzon J, Alver A, Ravikumar V, Devera A, Mikhael T, Nauman R, Simanton E (2021). The impact of educational resources and perceived preparedness on medical education performance. Med Sci Educ.

[5] Rauf A, Shamim MS, Aly SM, Chundrigar T, Alam SN (2014). Formative assessment in undergraduate medical education: concept, implementation and hurdles. J Pak Med Assoc.

[6] Norcini J, Anderson MB, Bollela V (2018). 2018 Consensus framework for good assessment. Med Teach.

[7] Singh T, Anshu Anshu, Modi JN (2012). The quarter model: a proposed approach for intraining assessment of undergraduate students in Indian medical schools. Indian Pediatr.

[8] Duffield KE, Spencer JA (2002). A survey of medical students' views about the purposes and fairness of assessment. Med Educ.

[9] Harris P, Snell L, Talbot M, Harden RM (2010). Competency-based medical education: implications for undergraduate programs. Med Teach.

[10] (2023). Formative Assessment: Pros and Cons You Need to Know. https://educationadvanced.com/resources/blog/formative-assessment-pros-and-cons/.

[11] Bawane J, Sharma R (2020). Formative Assessments and the Continuity of Learning During Emergencies and Crises. Published online.

[12] (2023). Complex adaptive systems overview. https://youtu.be/IWhkUne8T68.

[13] Matheson A, Dew K, Cumming J (2009). Complexity, evaluation and the effectiveness of community-based interventions to reduce health inequalities. Health Promot J Austr.

[14] Keshavarz N, Nutbeam D, Rowling L, Khavarpour F (2010). Schools as social complex adaptive systems: a new way to understand the challenges of introducing the health promoting schools concept. Soc Sci Med.

[15] Mash BJ, Mayers P, Conradie H, Orayn A, Kuiper M, Marais J (2008). How to manage organisational change and create practice teams: experiences of a South African primary care health centre. Educ Health (Abingdon).

[16] Anderson RA, Issel LM, McDaniel RR Jr (2003). Nursing homes as complex adaptive systems: relationship between management practice and resident outcomes. Nurs Res.

[17] (2022). 5 Principles for social sustainability. https://www.youtube.com/watch?v=o6lSuwJw0pk.

[18] Woodruff JN (2019). Accounting for complexity in medical education: a model of adaptive behaviour in medicine. Med Educ.

[19] McKenzie S (2023). Social sustainability: towards some definitions. Social sustainability: towards some definitions.

[20] Glatter R (2006). Leadership and organization in education: time for a re-orientation?1. Sch Leadersh Manag.

[21] Preston R, Gratani M, Owens K, Roche P, Zimanyi M, Malau-Aduli B (2020). Exploring the impact of assessment on medical students’ learning. Assess Eval High Educ.

[22] Hashmat S, Hashmat M, Amanullah F, Aziz S (2008). Factors causing exam anxiety in medical students. J Pak Med Assoc.

[23] Pelletier K, Brown M, Brooks DC, McCormack M, Reeves J, Arbino N (2021). 2021 EDUCAUSE Horizon Report: Teaching and Learning Edition. https://www.wested.org/online_pubs/resource1307.pdf.

[24] (2022). Identifying & addressing learning gaps. https://teaching.cornell.edu/identifying-addressing-learning-gaps.

[25] Parboteeah S, Anwar M (2009). Thematic analysis of written assignment feedback: implications for nurse education. Nurse Educ Today.

[26] Agius NM, Wilkinson A (2014). Students' and teachers' views of written feedback at undergraduate level: a literature review. Nurse Educ Today.

[27] Cockett A, Jackson C (2018). The use of assessment rubrics to enhance feedback in higher education: an integrative literature review. Nurse Educ Today.

[28] Gulikers JTM, Bastiaens TJ, Kirschner PA (2004). A five-dimensional framework for authentic assessment. Educ Technol Res Dev.

[29] Chaudhuri A, Adhya D (2019). A study to assess the effects of continuous weekly assessment along with providing feedback on the final performance in examination of first MBBS students in physiology. Int J Res Rev.

[30] Santra R, Pramanik S, Mandal A, Sengupta P, Das N, Raychaudhuri P (2014). A study on the performance of medical students in internal assessment and its correlates to final examinations of 2(nd) MBBS pharmacology curriculum in a medical college of eastern India. J Clin Diagn Res.

[31] Machado MB, Ribeiro DL, de Carvalho Filho MA (2022). Social justice in medical education: inclusion is not enough-it's just the first step. Perspect Med Educ.

